# *M*-type Gd_2_[Si_2_O_7_]

**DOI:** 10.1107/S2414314623006545

**Published:** 2023-08-01

**Authors:** Ralf Jules Christian Locke, Thomas Schleid

**Affiliations:** a Institut für Anorganische Chemie, Universität Stuttgart, Pfaffenwaldring 55, 70569 Stuttgart, Germany; Vienna University of Technology, Austria

**Keywords:** crystal structure, oxidodisilicate, rare-earth metal, gadolinium, isotypism

## Abstract

The title compound, digadolinium(III) oxidodisilicate, Gd_2_[Si_2_O_7_], was obtained in its *M*-type crystal structure after attempts to synthesize Gd_5_Br_3_[AsO_3_]_4_ as a by-product from fused silica ampoules. It crystallizes isotypically with *M*-type Eu_2_[Si_2_O_7_]. This structure consists of layers of ecliptically arranged oxidodisilicate [Si_2_O_7_]^6−^ units separated from each other by bilayers consisting of Gd^III^ cations.

## Abstract 2

*M*-type Gd_2_[Si_2_O_7_] crystallizes isotypically with Eu_2_[Si_2_O_7_].

## Graphical abstract

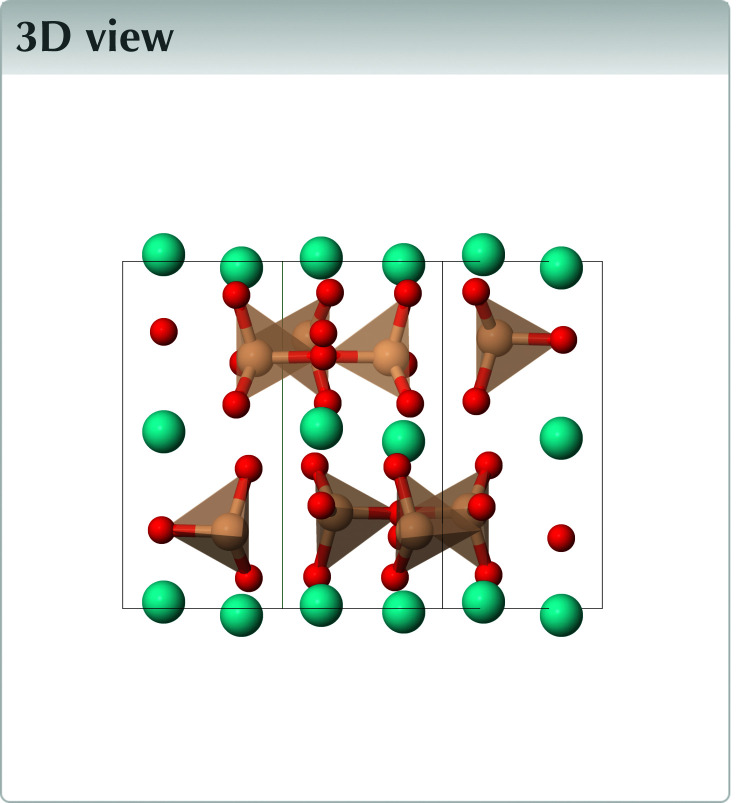

## Structure description

**Figure 1:**
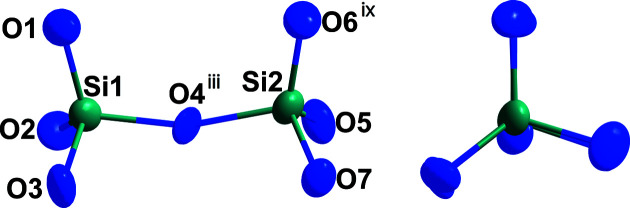
The unique oxidodisilicate [Si_2_O_7_]^6−^ anion composed of two vertex-connected [SiO_4_]^4−^ tetra­hedra in *M*-type Gd_2_[Si_2_O_7_], where the position of the O atoms define a horseshoe arrangement (left), and its Newman projection (right). Displacement ellipsoids are drawn at the 95% probability level. The symmetry codes are available in Table 1 ▸.

**Figure 2:**
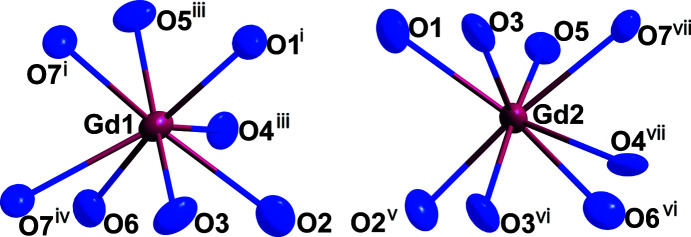
The oxygen environment of the two crystallographically different Gd^III^ cations in *M*-type Gd_2_[Si_2_O_7_]. Displacement ellipsoids are drawn at the 95% probability level. The symmetry codes are available in Table 1 ▸.

**Figure 3:**
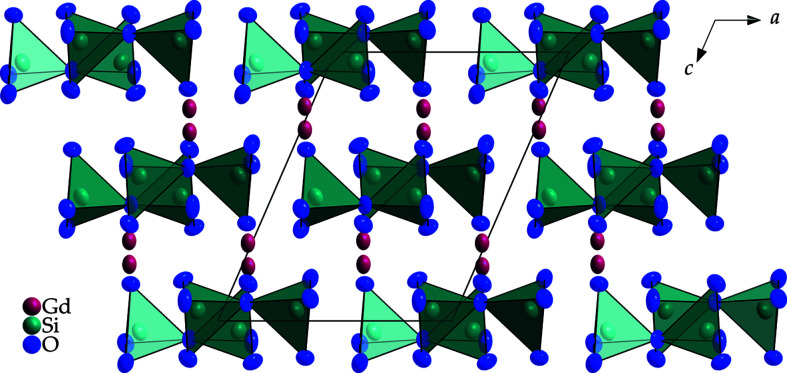
View at the monoclinic crystal structure of *M*-type Gd_2_[Si_2_O_7_] along [010] emphasizing the discrete [Si_2_O_7_]^6−^ anions (polyhedral representation). Displacement ellipsoids are drawn at the 95% probability level.

*M*-type Gd_2_[Si_2_O_7_] crystallizes as colourless platelets, isotypic with *M*-type Eu_2_[Si_2_O_7_] (Strobel *et al.*, 2009 ▸), in the monoclinic space group *P*2_1_/*n*. In the crystal structure, the two crystallographically distinguishable [SiO_4_]^4−^ tetra­hedra form discrete ecliptically arranged oxidodisilicate [Si_2_O_7_]^6−^ units, which stand only on ‘two legs’, here atoms O1 and O6, like the *E*- (Felsche, 1970 ▸) and ζ-type oxidodisilicates (Hartenbach *et al.*, 2006 ▸), leading to the so-called ‘horseshoe’ conformation, with an Si1—O4—Si2 angle of 161.3 (3)° (Fig. 1 ▸). Within the oxidosilicate tetra­hedra, Si—O distances (Table 1 ▸) ranging from 1.588 (6) to 1.639 (6) Å to the terminal, as well as 1.646 (8) and 1.662 (8) Å to the bridging, oxide ligands (O4) occur, which agree well with those of the well-known dieuropium(III) oxidodisilicate, Eu_2_[Si_2_O_7_], in its *M*-type structure [*d*(Si—O) = 1.61–1.66 Å] (Strobel *et al.*, 2009 ▸). Six terminal and four edge-bridging Gd^III^ cations coordinate to each vertex-shared [Si_2_O_7_]^6−^ bi­tetra­hedron, two of which bind one edge each of two terminal O^2−^ anions of one tetra­hedral half (O1—O3 and O6—O7) and two of which bind three times each to one terminal O^2−^ anion of both tetra­hedral halves of the oxosilicate doubles, as well as to the bridging O atom (O2⋯O4⋯O5 and O3⋯O4⋯O7). Both crystallographically distinct Gd^III^ cations are surrounded by eight O^2−^ anions, each with Gd—O distances ranging from 2.250 (8) to 2.691 (6) Å (Fig. 2 ▸). In the crystal structure of *M*-type Gd_2_[Si_2_O_7_], the [Si_2_O_7_]^6−^ units are present in a layered arrangement parallel to (001) with adjacent bi­tetra­hedra occurring mirrored along [010] at the inversion centre, whereas they are identically oriented along [100]. This structure consists of layers of [Si_2_O_7_]^6−^ units separated from each other by bilayers consisting of Gd^III^ cations (Fig. 3 ▸).

## Synthesis and crystallization

Single crystals of *M*-Gd_2_[Si_2_O_7_] were obtained as a by-product during the synthesis of Gd_5_Br_3_[AsO_3_]_4_ (Locke *et al.*, 2023 ▸) by reacting Gd_2_O_3_ with fused silica (SiO_2_) as the reaction vessel at a temperature of 1100 K, taking advantage of the presumed mineralizers As_2_O_3_ and GdBr_3_. The transparent colourless crystals exhibit a platelet-like habit.

## Refinement

Crystal data, data collection and structure refinement details are summarized in Table 2 ▸.

## Supplementary Material

Crystal structure: contains datablock(s) I, global. DOI: 10.1107/S2414314623006545/wm4193sup1.cif

Structure factors: contains datablock(s) I. DOI: 10.1107/S2414314623006545/wm4193Isup2.hkl

CCDC reference: 2279127

Additional supporting information:  crystallographic information; 3D view; checkCIF report

## Figures and Tables

**Table 1:** Selected bond lengths (Å)

Gd1—O1^i^	2.291 (5)	Gd2—O3	2.565 (7)
Gd1—O7^i^	2.305 (8)	Gd2—O4^vii^	2.655 (5)
Gd1—O3^ii^	2.376 (8)	Gd2—Si1	3.103 (3)
Gd1—O6	2.384 (5)	Gd2—Si2^vii^	3.233 (3)
Gd1—O5^iii^	2.441 (7)	Gd2—Si1^vi^	3.269 (3)
Gd1—O2	2.533 (8)	Gd2—Gd1^viii^	3.8078 (6)
Gd1—O4^iii^	2.621 (5)	Si1—O2	1.588 (6)
Gd1—O7^iv^	2.691 (6)	Si1—O1	1.612 (6)
Gd1—Si2^iv^	3.128 (3)	Si1—O3	1.635 (6)
Gd1—Si2^iii^	3.217 (3)	Si1—O4^iii^	1.646 (8)
Gd1—Si1	3.277 (3)	Si1—Gd2^vi^	3.269 (3)
Gd1—Gd2^iii^	3.8078 (6)	Si2—O5	1.595 (6)
Gd2—O5	2.250 (8)	Si2—O6^ix^	1.600 (6)
Gd2—O2^v^	2.302 (7)	Si2—O7	1.639 (6)
Gd2—O6^vi^	2.313 (5)	Si2—O4	1.662 (8)
Gd2—O7^vii^	2.471 (8)	Si2—Gd1^ix^	3.128 (3)
Gd2—O1	2.472 (5)	Si2—Gd1^viii^	3.217 (3)
Gd2—O3^vi^	2.522 (8)	Si2—Gd2^x^	3.233 (3)

Symmetry codes: (i) 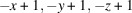 ; (ii) 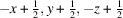 ; (iii) 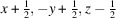 ; (iv) 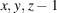 ; (v) 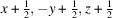 ; (vi) 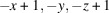 ; (vii) 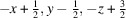 ; (viii) 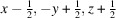 ; (ix) 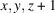 ; (x) 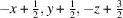 .

**Table 2:** Experimental details

Crystal data	
Chemical formula	Gd_2_[Si_2_O_7_]
*M*_r_	482.68
Crystal system, space group	Monoclinic, *P*2_1_/*n*
Temperature (K)	293
*a*, *b*, *c* (Å)	7.7267 (5), 8.3859 (6), 9.6814 (7)
β (°)	113.486 (3)
*V* (Å^3^)	575.34 (7)
*Z*	4
Radiation type	Mo *K*α
μ (mm^−1^)	23.25
Crystal size (mm)	1.0 × 0.5 × 0.1

Data collection	
Diffractometer	Stoe StadiVari
Absorption correction	Numerical (*LANA*; Koziskova *et al.*, 2016 ▸)
*T*_min_, *T*_max_	0.031, 0.108
No. of measured, independent and observed [*I* > 2σ(*I*)] reflections	11604, 2030, 1462
*R*_int_	0.051
(sin θ/λ)_max_ (Å^−1^)	0.766

Refinement	
*R*[*F* ^2^ > 2σ(*F* ^2^)], *wR*(*F* ^2^), *S*	0.033, 0.068, 0.95
No. of reflections	2030
No. of parameters	102
Δρ_max_, Δρ_min_ (e Å^−3^)	1.72, −1.87

Computer programs: *X-AREA* (Stoe & Cie, 2020 ▸), *SHELXT* (Sheldrick, 2015*a* ▸), *SHELXL* (Sheldrick, 2015*b* ▸), *DIAMOND* (Brandenburg & Putz, 2005 ▸) and *publCIF* (Westrip, 2010 ▸).

## full crystallographic data

### Crystal data

**Table:**

Gd_2_[Si_2_O_7_]	*F*(000) = 848
*M**_r_* = 482.68	*D*_x_ = 5.572 Mg m^−^^3^
Monoclinic, *P*2_1_/*n*	Mo *K*α radiation, λ = 0.71069 Å
*a* = 7.7267 (5) Å	Cell parameters from 12003 reflections
*b* = 8.3859 (6) Å	θ = 2.3–33.0°
*c* = 9.6814 (7) Å	µ = 23.25 mm^−^^1^
β = 113.486 (3)°	*T* = 293 K
*V* = 575.34 (7) Å^3^	Platelet, colourless
*Z* = 4	1.0 × 0.5 × 0.1 mm

### Data collection

**Table:**

Stoe StadiVari diffractometer	2030 independent reflections
Radiation source: fine-focus sealed tube	1462 reflections with *I* > 2σ(*I*)
Detector resolution: 5.81 pixels mm^-1^	*R*_int_ = 0.051
rotation method, ω scans	θ_max_ = 33.0°, θ_min_ = 2.9°
Absorption correction: numerical (*LANA*; Koziskova *et al.*, 2016)	*h* = −11→11
*T*_min_ = 0.031, *T*_max_ = 0.108	*k* = −12→12
11604 measured reflections	*l* = −14→14

### Refinement

**Table:**

Refinement on *F*^2^	0 restraints
Least-squares matrix: full	*w* = 1/[σ^2^(*F*_o_^2^) + (0.0305*P*)^2^] where *P* = (*F*_o_^2^ + 2*F*_c_^2^)/3
*R*[*F*^2^ > 2σ(*F*^2^)] = 0.033	(Δ/σ)_max_ < 0.001
*wR*(*F*^2^) = 0.068	Δρ_max_ = 1.72 e Å^−^^3^
*S* = 0.95	Δρ_min_ = −1.86 e Å^−^^3^
2030 reflections	Extinction correction: SHELXL (Sheldrick, 2015b), Fc^*^=kFc[1+0.001xFc^2^λ^3^/sin(2θ)]^-1/4^
102 parameters	Extinction coefficient: 0.0096 (3)

### Special details

**Table:**

Geometry. All esds (except the esd in the dihedral angle between two l.s. planes) are estimated using the full covariance matrix. The cell esds are taken into account individually in the estimation of esds in distances, angles and torsion angles; correlations between esds in cell parameters are only used when they are defined by crystal symmetry. An approximate (isotropic) treatment of cell esds is used for estimating esds involving l.s. planes.
Refinement. Refined as a 2-component twin.

### Fractional atomic coordinates and isotropic or equivalent isotropic displacement parameters (Å^2^)

**Table:**

	*x*	*y*	*z*	*U*_iso_*/*U*_eq_	
Gd1	0.48343 (8)	0.48090 (4)	0.21037 (4)	0.00929 (12)	
Gd2	0.47192 (8)	0.00947 (4)	0.70211 (4)	0.00892 (13)	
Si1	0.3936 (4)	0.2763 (3)	0.4626 (3)	0.0091 (6)	
Si2	0.3149 (4)	0.2212 (3)	0.9603 (3)	0.0098 (6)	
O1	0.4411 (13)	0.2968 (6)	0.6396 (6)	0.0128 (12)	
O2	0.2751 (11)	0.4101 (7)	0.3477 (7)	0.0126 (15)	
O3	0.3205 (12)	0.0922 (7)	0.4242 (7)	0.0140 (15)	
O4	0.0880 (10)	0.2262 (6)	0.9314 (6)	0.0118 (11)	
O5	0.3162 (11)	0.0874 (6)	0.8436 (7)	0.0109 (14)	
O6	0.4408 (13)	0.2074 (6)	0.1370 (6)	0.0125 (12)	
O7	0.3525 (12)	0.4030 (6)	0.9167 (7)	0.0104 (14)	

### Atomic displacement parameters (Å^2^)

**Table:**

	*U*^11^	*U*^22^	*U*^33^	*U*^12^	*U*^13^	*U*^23^
Gd1	0.0079 (2)	0.0081 (2)	0.0127 (2)	0.0003 (2)	0.0050 (2)	0.00029 (16)
Gd2	0.0078 (2)	0.0078 (2)	0.0120 (2)	−0.0004 (2)	0.0048 (2)	0.00001 (16)
Si1	0.0085 (14)	0.0073 (12)	0.0108 (12)	0.0016 (9)	0.0032 (10)	0.0000 (9)
Si2	0.0102 (14)	0.0087 (13)	0.0120 (12)	−0.0013 (9)	0.0060 (10)	0.0010 (9)
O1	0.014 (4)	0.014 (3)	0.012 (3)	−0.004 (4)	0.007 (4)	−0.001 (2)
O2	0.011 (4)	0.014 (3)	0.010 (3)	0.005 (3)	0.001 (3)	0.002 (2)
O3	0.009 (4)	0.014 (3)	0.021 (4)	−0.003 (3)	0.008 (3)	−0.003 (2)
O4	0.008 (3)	0.011 (3)	0.014 (3)	0.005 (3)	0.005 (3)	0.001 (2)
O5	0.009 (4)	0.009 (3)	0.014 (3)	0.000 (3)	0.004 (3)	−0.005 (2)
O6	0.012 (4)	0.017 (3)	0.009 (3)	0.000 (4)	0.004 (4)	−0.001 (2)
O7	0.012 (4)	0.006 (3)	0.014 (3)	−0.001 (3)	0.007 (3)	−0.001 (2)

### Geometric parameters (Å, º)

**Table:**

Gd1—O1^i^	2.291 (5)	Si1—O1	1.612 (6)
Gd1—O7^i^	2.305 (8)	Si1—O3	1.635 (6)
Gd1—O3^ii^	2.376 (8)	Si1—O4^iii^	1.646 (8)
Gd1—O6	2.384 (5)	Si1—Gd2^vi^	3.269 (3)
Gd1—O5^iii^	2.441 (7)	Si2—O5	1.595 (6)
Gd1—O2	2.533 (8)	Si2—O6^ix^	1.600 (6)
Gd1—O4^iii^	2.621 (5)	Si2—O7	1.639 (6)
Gd1—O7^iv^	2.691 (6)	Si2—O4	1.662 (8)
Gd1—Si2^iv^	3.128 (3)	Si2—Gd1^ix^	3.128 (3)
Gd1—Si2^iii^	3.217 (3)	Si2—Gd1^viii^	3.217 (3)
Gd1—Si1	3.277 (3)	Si2—Gd2^x^	3.233 (3)
Gd1—Gd2^iii^	3.8078 (6)	O1—Gd1^i^	2.291 (5)
Gd2—O5	2.250 (8)	O2—Gd2^xi^	2.302 (7)
Gd2—O2^v^	2.302 (7)	O3—Gd1^xii^	2.376 (8)
Gd2—O6^vi^	2.313 (5)	O3—Gd2^vi^	2.522 (8)
Gd2—O7^vii^	2.471 (8)	O4—Si1^viii^	1.646 (8)
Gd2—O1	2.472 (5)	O4—Gd1^viii^	2.621 (5)
Gd2—O3^vi^	2.522 (8)	O4—Gd2^x^	2.655 (5)
Gd2—O3	2.565 (7)	O5—Gd1^viii^	2.441 (7)
Gd2—O4^vii^	2.655 (5)	O6—Si2^iv^	1.600 (6)
Gd2—Si1	3.103 (3)	O6—Gd2^vi^	2.313 (5)
Gd2—Si2^vii^	3.233 (3)	O7—Gd1^i^	2.305 (8)
Gd2—Si1^vi^	3.269 (3)	O7—Gd2^x^	2.471 (8)
Gd2—Gd1^viii^	3.8078 (6)	O7—Gd1^ix^	2.691 (6)
Si1—O2	1.588 (6)		
			
O1^i^—Gd1—O7^i^	86.5 (3)	O5—Gd2—Si2^vii^	94.83 (17)
O1^i^—Gd1—O3^ii^	88.5 (3)	O2^v^—Gd2—Si2^vii^	148.23 (16)
O7^i^—Gd1—O3^ii^	100.09 (19)	O6^vi^—Gd2—Si2^vii^	72.48 (17)
O1^i^—Gd1—O6	160.3 (2)	O7^vii^—Gd2—Si2^vii^	29.74 (15)
O7^i^—Gd1—O6	106.7 (3)	O1—Gd2—Si2^vii^	128.99 (19)
O3^ii^—Gd1—O6	103.0 (3)	O3^vi^—Gd2—Si2^vii^	87.49 (15)
O1^i^—Gd1—O5^iii^	84.7 (3)	O3—Gd2—Si2^vii^	75.78 (15)
O7^i^—Gd1—O5^iii^	72.1 (2)	O4^vii^—Gd2—Si2^vii^	30.84 (16)
O3^ii^—Gd1—O5^iii^	170.0 (2)	Si1—Gd2—Si2^vii^	106.49 (7)
O6—Gd1—O5^iii^	85.6 (3)	O5—Gd2—Si1^vi^	149.41 (16)
O1^i^—Gd1—O2	85.0 (3)	O2^v^—Gd2—Si1^vi^	94.01 (18)
O7^i^—Gd1—O2	168.21 (19)	O6^vi^—Gd2—Si1^vi^	71.81 (19)
O3^ii^—Gd1—O2	71.6 (2)	O7^vii^—Gd2—Si1^vi^	88.20 (16)
O6—Gd1—O2	83.7 (3)	O1—Gd2—Si1^vi^	127.82 (19)
O5^iii^—Gd1—O2	115.06 (16)	O3^vi^—Gd2—Si1^vi^	29.34 (15)
O1^i^—Gd1—O4^iii^	95.96 (18)	O3—Gd2—Si1^vi^	77.72 (16)
O7^i^—Gd1—O4^iii^	131.3 (2)	O4^vii^—Gd2—Si1^vi^	30.05 (17)
O3^ii^—Gd1—O4^iii^	128.6 (2)	Si1—Gd2—Si1^vi^	99.74 (6)
O6—Gd1—O4^iii^	64.33 (18)	Si2^vii^—Gd2—Si1^vi^	60.26 (5)
O5^iii^—Gd1—O4^iii^	59.7 (2)	O5—Gd2—Gd1^viii^	37.46 (17)
O2—Gd1—O4^iii^	58.0 (2)	O2^v^—Gd2—Gd1^viii^	139.14 (19)
O1^i^—Gd1—O7^iv^	139.54 (18)	O6^vi^—Gd2—Gd1^viii^	91.6 (3)
O7^i^—Gd1—O7^iv^	66.0 (3)	O7^vii^—Gd2—Gd1^viii^	35.64 (16)
O3^ii^—Gd1—O7^iv^	69.1 (3)	O1—Gd2—Gd1^viii^	89.1 (2)
O6—Gd1—O7^iv^	60.18 (17)	O3^vi^—Gd2—Gd1^viii^	147.64 (15)
O5^iii^—Gd1—O7^iv^	112.0 (2)	O3—Gd2—Gd1^viii^	88.6 (2)
O2—Gd1—O7^iv^	116.6 (2)	O4^vii^—Gd2—Gd1^viii^	92.70 (16)
O4^iii^—Gd1—O7^iv^	124.43 (16)	Si1—Gd2—Gd1^viii^	95.78 (5)
O1^i^—Gd1—Si2^iv^	168.31 (19)	Si2^vii^—Gd2—Gd1^viii^	62.16 (5)
O7^i^—Gd1—Si2^iv^	91.68 (16)	Si1^vi^—Gd2—Gd1^viii^	122.43 (5)
O3^ii^—Gd1—Si2^iv^	80.41 (16)	O2—Si1—O1	119.5 (4)
O6—Gd1—Si2^iv^	30.06 (14)	O2—Si1—O3	117.2 (4)
O5^iii^—Gd1—Si2^iv^	105.74 (15)	O1—Si1—O3	104.7 (3)
O2—Gd1—Si2^iv^	95.01 (15)	O2—Si1—O4^iii^	101.2 (4)
O4^iii^—Gd1—Si2^iv^	93.91 (12)	O1—Si1—O4^iii^	111.0 (4)
O7^iv^—Gd1—Si2^iv^	31.58 (13)	O3—Si1—O4^iii^	101.8 (4)
O1^i^—Gd1—Si2^iii^	91.52 (18)	O2—Si1—Gd2	156.8 (3)
O7^i^—Gd1—Si2^iii^	100.47 (18)	O1—Si1—Gd2	52.31 (19)
O3^ii^—Gd1—Si2^iii^	159.41 (19)	O3—Si1—Gd2	55.7 (2)
O6—Gd1—Si2^iii^	72.03 (17)	O4^iii^—Si1—Gd2	102.0 (2)
O5^iii^—Gd1—Si2^iii^	28.81 (16)	O2—Si1—Gd2^vi^	112.5 (3)
O2—Gd1—Si2^iii^	87.91 (15)	O1—Si1—Gd2^vi^	127.9 (3)
O4^iii^—Gd1—Si2^iii^	30.98 (17)	O3—Si1—Gd2^vi^	49.1 (3)
O7^iv^—Gd1—Si2^iii^	121.22 (15)	O4^iii^—Si1—Gd2^vi^	53.89 (19)
Si2^iv^—Gd1—Si2^iii^	100.16 (3)	Gd2—Si1—Gd2^vi^	80.26 (6)
O1^i^—Gd1—Si1	91.69 (18)	O2—Si1—Gd1	48.9 (3)
O7^i^—Gd1—Si1	160.70 (18)	O1—Si1—Gd1	135.2 (3)
O3^ii^—Gd1—Si1	99.08 (19)	O3—Si1—Gd1	118.8 (3)
O6—Gd1—Si1	70.92 (19)	O4^iii^—Si1—Gd1	52.41 (19)
O5^iii^—Gd1—Si1	88.55 (16)	Gd2—Si1—Gd1	153.91 (9)
O2—Gd1—Si1	28.19 (15)	Gd2^vi^—Si1—Gd1	79.48 (6)
O4^iii^—Gd1—Si1	29.85 (17)	O5—Si2—O6^ix^	122.3 (4)
O7^iv^—Gd1—Si1	123.92 (15)	O5—Si2—O7	114.8 (4)
Si2^iv^—Gd1—Si1	93.76 (7)	O6^ix^—Si2—O7	104.4 (3)
Si2^iii^—Gd1—Si1	60.34 (5)	O5—Si2—O4	101.7 (4)
O1^i^—Gd1—Gd2^iii^	89.6 (3)	O6^ix^—Si2—O4	109.5 (4)
O7^i^—Gd1—Gd2^iii^	38.66 (17)	O7—Si2—O4	102.5 (4)
O3^ii^—Gd1—Gd2^iii^	138.72 (18)	O5—Si2—Gd1^ix^	156.8 (3)
O6—Gd1—Gd2^iii^	92.0 (2)	O6^ix^—Si2—Gd1^ix^	48.29 (18)
O5^iii^—Gd1—Gd2^iii^	34.10 (16)	O7—Si2—Gd1^ix^	59.3 (2)
O2—Gd1—Gd2^iii^	149.15 (15)	O4—Si2—Gd1^ix^	101.5 (2)
O4^iii^—Gd1—Gd2^iii^	92.60 (17)	O5—Si2—Gd1^viii^	47.5 (3)
O7^iv^—Gd1—Gd2^iii^	86.64 (19)	O6^ix^—Si2—Gd1^viii^	136.8 (3)
Si2^iv^—Gd1—Gd2^iii^	96.26 (5)	O7—Si2—Gd1^viii^	117.8 (3)
Si2^iii^—Gd1—Gd2^iii^	61.86 (5)	O4—Si2—Gd1^viii^	54.3 (2)
Si1—Gd1—Gd2^iii^	122.19 (5)	Gd1^ix^—Si2—Gd1^viii^	155.51 (9)
O5—Gd2—O2^v^	101.69 (19)	O5—Si2—Gd2^x^	112.2 (3)
O5—Gd2—O6^vi^	84.4 (3)	O6^ix^—Si2—Gd2^x^	125.5 (3)
O2^v^—Gd2—O6^vi^	82.3 (3)	O7—Si2—Gd2^x^	48.4 (3)
O5—Gd2—O7^vii^	72.5 (2)	O4—Si2—Gd2^x^	55.0 (2)
O2^v^—Gd2—O7^vii^	170.8 (2)	Gd1^ix^—Si2—Gd2^x^	81.32 (6)
O6^vi^—Gd2—O7^vii^	89.9 (3)	Gd1^viii^—Si2—Gd2^x^	80.91 (6)
O5—Gd2—O1	81.0 (3)	Si1—O1—Gd1^i^	130.8 (3)
O2^v^—Gd2—O1	80.8 (2)	Si1—O1—Gd2	96.6 (2)
O6^vi^—Gd2—O1	154.8 (2)	Gd1^i^—O1—Gd2	131.5 (3)
O7^vii^—Gd2—O1	104.9 (3)	Si1—O2—Gd2^xi^	142.9 (5)
O5—Gd2—O3^vi^	172.35 (19)	Si1—O2—Gd1	102.9 (4)
O2^v^—Gd2—O3^vi^	73.0 (2)	Gd2^xi^—O2—Gd1	108.3 (2)
O6^vi^—Gd2—O3^vi^	89.4 (3)	Si1—O3—Gd1^xii^	132.2 (4)
O7^vii^—Gd2—O3^vi^	112.04 (17)	Si1—O3—Gd2^vi^	101.6 (4)
O1—Gd2—O3^vi^	103.2 (3)	Gd1^xii^—O3—Gd2^vi^	106.4 (2)
O5—Gd2—O3	115.4 (3)	Si1—O3—Gd2	92.6 (3)
O2^v^—Gd2—O3	119.4 (2)	Gd1^xii^—O3—Gd2	114.0 (3)
O6^vi^—Gd2—O3	143.85 (18)	Gd2^vi^—O3—Gd2	107.8 (3)
O7^vii^—Gd2—O3	69.9 (3)	Si1^viii^—O4—Si2	161.3 (3)
O1—Gd2—O3	61.35 (18)	Si1^viii^—O4—Gd1^viii^	97.7 (3)
O3^vi^—Gd2—O3	72.2 (3)	Si2—O4—Gd1^viii^	94.7 (3)
O5—Gd2—O4^vii^	121.6 (2)	Si1^viii^—O4—Gd2^x^	96.1 (3)
O2^v^—Gd2—O4^vii^	119.9 (2)	Si2—O4—Gd2^x^	94.2 (3)
O6^vi^—Gd2—O4^vii^	64.64 (16)	Gd1^viii^—O4—Gd2^x^	104.97 (18)
O7^vii^—Gd2—O4^vii^	60.2 (2)	Si2—O5—Gd2	142.3 (4)
O1—Gd2—O4^vii^	140.50 (18)	Si2—O5—Gd1^viii^	103.7 (4)
O3^vi^—Gd2—O4^vii^	58.9 (2)	Gd2—O5—Gd1^viii^	108.4 (2)
O3—Gd2—O4^vii^	79.25 (17)	Si2^iv^—O6—Gd2^vi^	131.8 (3)
O5—Gd2—Si1	104.65 (16)	Si2^iv^—O6—Gd1	101.7 (3)
O2^v^—Gd2—Si1	95.40 (16)	Gd2^vi^—O6—Gd1	126.0 (2)
O6^vi^—Gd2—Si1	171.0 (2)	Si2—O7—Gd1^i^	136.3 (4)
O7^vii^—Gd2—Si1	93.06 (15)	Si2—O7—Gd2^x^	101.8 (4)
O1—Gd2—Si1	31.07 (14)	Gd1^i^—O7—Gd2^x^	105.7 (2)
O3^vi^—Gd2—Si1	81.58 (15)	Si2—O7—Gd1^ix^	89.1 (3)
O3—Gd2—Si1	31.75 (14)	Gd1^i^—O7—Gd1^ix^	114.0 (3)
O4^vii^—Gd2—Si1	109.70 (12)	Gd2^x^—O7—Gd1^ix^	106.8 (3)

Symmetry codes: (i) −*x*+1, −*y*+1, −*z*+1; (ii) −*x*+1/2, *y*+1/2, −*z*+1/2; (iii) *x*+1/2, −*y*+1/2, *z*−1/2; (iv) *x*, *y*, *z*−1; (v) *x*+1/2, −*y*+1/2, *z*+1/2; (vi) −*x*+1, −*y*, −*z*+1; (vii) −*x*+1/2, *y*−1/2, −*z*+3/2; (viii) *x*−1/2, −*y*+1/2, *z*+1/2; (ix) *x*, *y*, *z*+1; (x) −*x*+1/2, *y*+1/2, −*z*+3/2; (xi) *x*−1/2, −*y*+1/2, *z*−1/2; (xii) −*x*+1/2, *y*−1/2, −*z*+1/2.
